# Lectin-Based Antiviral Strategies for Porcine Reproductive and Respiratory Syndrome Virus 2 Infection: Griffithsin Suppresses Viral Replication In Vitro and Reduces Early Viremia In Vivo

**DOI:** 10.3390/microorganisms14051098

**Published:** 2026-05-12

**Authors:** Darshana Kadekar, Deepak Velayudhan, Ester Vinyeta, Jianqiang Zhang, Ethan Aljets, Veeraya Bamrung, Panchan Sitthicharoenchai, Alyona Michael, Keith Frogue, Meng Heng, Amy Liu, Cristina Bongiorni, Manasi Bhate, David A. Estell, Chong Shen, Charlotte Poulsen

**Affiliations:** 1 Gut Immunology Lab, Research and Development, Health & Biosciences, International Flavors & Fragrances Inc., Edwin Rahrs Vej 38, 8220 Brabrand, Denmark; 2 Fujifilm Biotechnologies, Biotek Alle 1, 3400 Hillerød, Denmark; 3 Danisco Animal Nutrition & Health, International Flavors & Fragrances Inc., 2340 Oegstgeest, The Netherlands; deepak.evelayudhan@iff.com (D.V.); ester.vinyeta@iff.com (E.V.); 4 College of Veterinary Medicine, Iowa State University, 1800 Christensen Drive, Ames, IA 50011, USA; jqzhang@iastate.edu (J.Z.); ealjets@iastate.edu (E.A.); veerayab@iastate.edu (V.B.); psitthi@ncsu.edu (P.S.); avm0031@auburn.edu (A.M.); 5 Department of Bioinformatics and Computational Biology, Iowa State University, Ames, IA 50011, USA; 6 College of Veterinary Medicine, North Carolina State University, Raleigh, NC 27607, USA; 7 College of Veterinary Medicine, Auburn University, 1130 Wire Road, Auburn, AL 36849, USA; 8 Research and Development, Health & Biosciences, International Flavors & Fragrances Inc., 925 Page Mill Road, Palo Alto, CA 94304, USA; keith.frogue@iff.com (K.F.); meng.heng@iff.com (M.H.); amy.liu-1@iff.com (A.L.); cristina.bongiorni@iff.com (C.B.); manasi.p.bhate@iff.com (M.B.); dave.estell@iff.com (D.A.E.)

**Keywords:** griffithsin (GRFT), intranasal antiviral delivery, lectin-based antivirals, porcine reproductive and respiratory syndrome virus (PRRSV), MARC-145 cells, swine health

## Abstract

Porcine reproductive and respiratory syndrome virus (PRRSV) remains a major challenge to swine production worldwide. Current vaccines have limited efficacy against genetically diverse PRRSV strains. Therefore, strategies with alternative modes of action—such as antiviral approaches that target conserved virus–host interactions, including viral attachment and entry, rather than relying solely on adaptive immune responses—are needed. We first evaluated the in vitro effect of griffithsin (GRFT), a high-mannose-binding lectin, in the monkey kidney cell line MARC-145. Cells were pre-treated with GRFT (50–200 µg/mL) prior to PRRSV infection, after which cell morphology and viral RNA replication (measured by RT-qPCR) were assessed. Pre-treatment with 100–200 µg/mL GRFT, followed by PRRSV inoculation at a multiplicity of infection of 1 or 10, reduced viral replication in MARC145 cells in a dose-dependent manner, achieving almost 100% inhibition of ORF5 and ORF7 RNA compared with untreated controls (*p* < 0.0001). We next investigated the in vivo effects of intranasal GRFT administration (7.5 or 15 mg/day) in pigs (*n* = 56). Pigs treated with 15 mg/day GRFT exhibited significantly reduced (*p* < 0.05) viremia 2, 4 and 7 days post-challenge, compared with untreated, challenged, and controls (log_10_ 8.1 ± 0.2 vs. 9.0 ± 0.25, 8.2 ± 0.1 vs. 9.1 ± 0.2, and 8.9 ± 0.2 vs. 9.3 ± 0.2, respectively), along with earlier resolution of fever and a trend toward increased average daily gain over 42 days (*p* < 0.1). These findings are the first report of GRFT efficacy in pigs and support its potential as an antiviral strategy against PRRSV, alongside existing interventions.

## 1. Introduction

Porcine reproductive and respiratory syndrome virus (PRRSV) remains one of the most economically consequential pathogens in global pork production. Since its emergence in the late 1980s and early 1990s, the virus has spread worldwide, with only a small proportion of countries—representing approximately 7% of the global pig population—remaining PRRSV-free [1]. PRRSV infection is associated with a broad spectrum of clinical and production outcomes, ranging from subclinical infection to severe reproductive failure in breeding animals and respiratory disease in growing–finishing pigs, which may lead to reduced feed intake, reduced weight gain, impaired feed efficiency, increased mortality, and high veterinary costs [1,2]. Certain strains of PRRSV may also cause brain infection [3,4]. PRRSV infection further disrupts immune homeostasis, increasing susceptibility to opportunistic pathogens and exacerbating disease burden [2,5].

Despite more than three decades of vaccine availability, PRRSV control remains challenging, mainly because current vaccines offer only limited cross-protection against genetically and antigenically diverse PRRSV strains [6,7,8]. Studies demonstrate that circulating PRRSV variants exhibit persistent infection and atypical seasonal transmission patterns, all of which complicate herd stabilization and viral elimination efforts [9,10,11]. These characteristics, together with the virus’s capacity to exploit multiple transmission routes (both direct via pig-to-pig contact and venereal transmission and indirect spread via fomites, aerosols, transportation vehicles, and personnel movement) and inconsistencies in on-farm biosecurity implementation, result in recurrent outbreaks even in well-managed farming systems [12]. Beyond animal health, PRRSV infection has been linked to greater environmental and resource use pressures in affected production systems, including emissions, land use, and nutrient efficiency [2]. In addition, PRRSV outbreaks have been associated with production losses and increased volatility in pork production systems, highlighting the persistent economic impact of the virus [2,13]. Over the past three decades, persistent limitations in PRRSV control—including incomplete vaccine cross-protection and continued viral circulation—have driven research on viral genetics, host–pathogen interactions, epidemiology, and host resilience to identify and prioritize targets for intervention. Collaborative initiatives such as the North American PRRS Research Consortium and the PRRS Host Genetics Consortium have reported associations between specific host genetic loci and variation in PRRSV viral load and production-related traits under experimental and field infection conditions [14,15]. Genetic strategies that modify PRRSV host cell entry factors—such as targeted editing of the host cell surface receptor CD163—have shown promise in in vivo studies without impairing growth or production traits; Nesbitt et al. demonstrated that pigs lacking the SRCR5 domain of CD163 exhibited heritable resistance to PRRSV [16]. Despite these advances, PRRSV remains challenging to control due to its rapid evolution and its capacity to subvert host innate immune pathways. While PRRSV has been shown to suppress DNA-sensing pathways such as cGAS–STING signaling [17], the virus is primarily detected through host RNA-sensing mechanisms. Viral genomic and replicative RNA can be recognized by cytosolic pattern-recognition receptors such as RIG-I and MDA5, as well as endosomal Toll-like receptors, including TLR3, TLR7, and TLR8, leading to activation of MAVS-, IRF3/7-, and NF-κB-dependent type-I interferon responses [18,19,20]. PRRSV has evolved multiple strategies to modulate these RNA-sensing pathways, including interference with RIG-I/MDA5 signaling and suppression of downstream interferon induction by several viral non-structural proteins [20,21,22]. Together, these immune evasion mechanisms highlight the need for antiviral interventions that operate independently of host genetics or traditional vaccine approaches.

C-type lectins (CTLs), a superfamily of glycan-binding receptors, have emerged as potentially encouraging antiviral and immunomodulatory targets in veterinary species due to their roles in glycan-dependent viral recognition, innate immune activation, and modulation of downstream adaptive response pathways [23]. As a lectin family, CTLs recognize conserved carbohydrate motifs on pathogens, enabling functions that include direct pathogen neutralization, inhibition of microbial adhesion to host cells, enhancement of phagocytosis, and regulation of inflammatory signaling pathways [24]. Beyond viral infections, lectins have been shown to modulate host responses to bacteria, fungi, and parasites through glycan-dependent interactions that influence pathogen clearance and immune cell activation [23]. In addition, exogenous glycan-binding lectins can disrupt viral attachment, entry, and cell-to-cell spread by targeting conserved glycans on viral envelope glycoproteins in experimental systems [25].

The red algal lectin griffithsin (GRFT), a high-mannose-binding lectin originally isolated from Griffithsia spp., has demonstrated potent and broad-spectrum antiviral activity against multiple enveloped viruses, including HIV-1 [26,27]; coronaviruses such as SARS-CoV, MERS-CoV, and SARS-CoV-2 [28,29]; influenza viruses [28]; filoviruses, including Ebola virus [28]; and PRRSV [30]. Mechanistically, GRFT exerts its antiviral activity through high-affinity multivalent binding to N-linked high-mannose glycans on viral envelope glycoproteins, thereby sterically blocking viral attachment to host cell receptors and preventing membrane fusion and viral entry [27,29]. GRFT has also been reported to interfere with cell-to-cell viral transmission and enhance viral neutralization through glycan clustering on virions [27,29]. Collectively, these findings highlight lectin-mediated targeting of conserved glycan structures as a mechanistically relevant strategy for controlling pathogens at the earliest stages of host interaction.

Despite its potent antiviral activity, native GRFT derived from red algae exhibits limitations common to lectin-based biologics, including susceptibility to proteolytic degradation and biophysical stability constraints [27,29], as well as challenges related to scalable and consistent manufacturability from natural sources and formulation for mucosal delivery [28,29,31]. Recent advances have expanded the landscape of glycan-targeting antiviral modalities. A 2024 patent (WO 2024/064653 A1) from our research group described engineered antiviral polypeptides, including lectin-based constructs and GRFT-referencing sequences, designed to modulate glycan-interacting antiviral mechanisms across host species [32]. Although not PRRSV-specific, this example illustrates the growing potential of engineered lectin-like molecules as antiviral agents and helped inform the rationale for our further evaluation of glycan-targeting lectins in the PRRSV infection model examined in the present study.

Given the substantial global burden of PRRSV, the incomplete protection offered by current interventions, and the mechanistic relevance of early virus–glycan interactions, we sought to explore CTLs, like GRFT, as an antiviral strategy targeting the earliest stages of infection. We hypothesized that GRFT may modulate early PRRSV infection processes in vitro and in vivo—particularly by impairing viral attachment and entry—and that such modulation could alter downstream innate immune signaling in infected cells, leading to reduced viremia and earlier resolution of PRRS-related symptoms in young pigs. This study aimed to (i) characterize the effects of a selected GRFT variant on PRRSV adsorption and early replication dynamics, and (ii) determine whether GRFT-mediated interference with viral attachment reduces overall infection efficiency and promotes growth performance in piglets. The objective was to determine whether a lectin-based antiviral approach based on GRFT could have potential for use in the control of PRRSV in young pigs.

## 2. Materials and Methods

### 2.1. Reagents and Materials

All cell culture media, equipment, and reagents were purchased from Thermo Fisher Scientific (Roskilde, Denmark), unless otherwise stated.

### 2.2. GRFT Preparation

The GRFT dimer variant S010N/G053K/N078Y, in which the two monomers are connected by a GTG linker, was selected as the engineered recombinant GRFT due to its enhanced protease stability, thermal stability, oxidative stability, and expression compared to the natural isolate. The mutant GRFT dimer gene was codon-optimized and heterologous expressed in *Bacillus subtilis*, as described in Patent Application WO2023/183865 [32]. Specifically, the S010N, G053K, and N078Y mutations were shown to confer enhanced resistance to degradation by native *Bacillus subtilis* proteases present in the expression strain.

The *Bacillus subtilis* culture was fermented under controlled conditions, and the secreted protein was recovered and purified using methods detailed in Patent Application WO2023/183865 [32]. The recombinant *B. subtilis* strain lacked five major proteases (AprE, NprE, Epr, IspA, and Bpr) and was engineered to introduce a start codon (ATG; methionine) at the N-terminus of the mature protein sequence. In the mature protein, the initiator methionine is post-translationally removed from the N-terminus, so residue numbering starts at the following serine, as shown in the sequence below:

SLTHRKFGG NGGSPFSGLS SIAVRSGSYL DAIIIDGVHH GGSGGNLSPT FTFKSGEYIS NMTIRSGDYI DNISFETNYG RRFGPYGGSG GSANTLSNVK VIQINGSAGD YLDSLDIYYE QYGTGSLTHR KFGGNGGSPF SGLSSIAVRS GSYLDAIIID GVHHGGSGGN LSPTFTFKSG EYISNMTIRS GDYIDNISFE TNYGRRFGPY GGSGGSANTL SNVKVIQING SAGDYLDSLD IYYEQY*.

### 2.3. Virus Propagation in MARC-145 Cells

Monkey kidney MARC-145 cells (purchased from IZSLER, Brescia, BS, Italy) were maintained in minimum essential medium (MEM) (supplemented with 10% fetal bovine serum (FBS), 1% penicillin–streptomycin (PS; containing 100 units (U)/mL penicillin and 100 μg/mL streptomycin), and L-glutamine. All cultures were incubated at 37 °C in a humidified atmosphere containing 5% CO_2_.

PRRSV-2 isolate DK-2010-10-13-1 (Gen Bank accession KF183946), a well-characterized field isolate that belongs to sublineage L5A according to the recent ORF5-based classification system [33], was kindly donated by Dr. Lars Erik Larsen (University of Copenhagen, Copenhagen, Denmark). For virus propagation, MARC-145 cells were seeded in T-175 flasks at a density of 1.5 × 10^7^ cells per flask in MEM supplemented with 5% FBS. Cultures were incubated overnight to achieve approximately 90% confluence. Cells were infected with the virus at a multiplicity of infection (MOI) of 0.03. The inoculum was prepared in 2% MEM, and 5 mL of the diluted virus was added per flask. Flasks were incubated for 60 min at 37 °C with gentle rocking every 10 min to ensure uniform viral distribution.

Following adsorption, 20 mL of 2% MEM was added to the flasks, and these were returned to the incubator. Cytopathic effects (CPE)—characterized by cell rounding and detachment—were monitored. Once >95% CPE had developed, flasks were tapped gently to detach remaining infected cells from the flask wall and immediately frozen at −80 °C.

For cell harvesting, frozen flasks were thawed and transferred to ice. The content of the entire flask was collected into a 50 mL conical tube and centrifuged at 3000× *g* for 1 h at 4 °C to remove cellular debris. The clarified supernatant was transferred to fresh tubes, and the total volume was recorded prior to further concentration. For virus concentration, the clarified supernatants were centrifuged at 20,000× *g* for 4 h at 4 °C, ensuring that the tubes were oriented so that the pellet formed on the marked side of the tube. Pellets were gently resuspended in T-1785 flasks containing 75 µL of 2% MEM. Aliquots (20 µL) of the concentrated virus were stored at −80 °C.

Virus titration was conducted in MARC-145 cells by plaque assay. Briefly, MARC-145 cells were seeded into 60 mm dishes in 5 mL of MEM + 5% FBS at a density of 0.8 × 10^6^ cells per dish. Dishes were incubated overnight at 37 °C to reach approximately 90% confluence. Serial 10-fold dilutions of virus (PRRSV-2 isolate DK-2010-10-13-1, 10^−1^ to 10^−10^) were prepared in 2% MEM on ice, using fresh pipette tips for each dilution. The growth medium (MEM) was removed, and cells were inoculated with 400 µL of 2% MEM mixed with 100 µL of the diluted virus. Plates were incubated for 1 h at 37 °C with gentle rocking every 15 min. After adsorption, 5 mL of overlay medium (2% MEM + 0.2% IgG) was added. Plates were incubated for 2–3 days until viral plaques became visible. Typically, plaques appeared at dilutions of around 10^−6^ to 10^−7^. For staining and enumeration, the growth medium was aspirated carefully, and 3 mL of 0.03% methylene blue solution was added for 5 min. Excess stain was removed, and the plaques were counted to compute the viral titer. Virus titers (PFU/mL) were calculated according to the following formula:

PFU/mL = Average plaques per dilution × 10/Dilution factor

### 2.4. Evaluation of GRFT Effects on PRRSV Infection in MARC-145 Cells

MARC-145 cells were seeded in 96-well cell culture plates one day before infection. The GRFT (50, 100 and 200 ug/mL) and virus isolate DK-2010-10-13-1 (MOI 1 and 10) were mixed and incubated at room temperature for 30 min. Then, the GRFT–virus mixture was added directly to the cells. Wells containing confluent MARC-145 cells in culture medium without GRFT–virus treatment served as the negative control, while only virus inoculation served as a positive control. After incubation, the unbound virus was washed off, fresh medium (MEM) was added, and the cells were further incubated for 24 h under the same conditions as before. The infected cells were examined under bright-field microscopy to observe cytopathic changes. Cells were then washed and lysed for the isolation of viral genomic RNA. Total RNA extraction and RT-qPCR were performed according to the method described by Li et al. [20] to quantify viral RNA. Primers targeting ORF5 and ORF7 were used. These are two genomic regions commonly used for molecular detection and characterization of PRRSV due to their diagnostic and epidemiological relevance. Host GAPDH mRNA served as the internal reference gene for normalization. Gene-specific primers were designed against conserved regions and validated through melt-curve analysis and gel electrophoresis to confirm specificity. The sequences of forward and reverse primers and probes for ORF5 and ORF7 are given in Supplementary Table S1.

The qPCR reactions were performed in 20 µL volumes containing SYBR Green master mix, 250 nM of each primer, and 2 µL of cDNA template. Thermal cycling was conducted under the following conditions: 95 °C for 2 min, followed by 40 cycles of 95 °C for 15 s and 60 °C for 30 s. A melt-curve step was included at the end of each run. All reactions were performed in technical triplicate, and any Ct values with an intra-triplicate SD > 0.3 cycles were discarded and the reactions repeated.

Normalization and relative quantification were performed according to the ΔCt method. For each sample, the threshold cycle (Ct) values of ORF5, ORF7, and GAPDH were obtained. Viral RNA levels were normalized to the host reference gene. Six technical replicates were averaged before ΔCt calculation.

Inhibition of PRRSV by GRFT in the GRFT-treated wells compared to the control wells without GRFT was calculated accordingly.

Each experimental condition was replicated 6 times.

### 2.5. GRFT Nasal Spray Preparation

A nasal spray of GRFT was prepared for use in an in vivo trial with young pigs. To prepare the nasal spray, 50 g of Methocel E3 Premium LV HP MC was dissolved in phosphate-buffered saline (PBS) to a total volume of 500 mL, resulting in a final concentration of 10% (*w*/*v*). Purified GRFT (Strain code KF474) was diluted in PBS at a concentration of 16.7 g/L (high dose) and 8.35 g/L (low dose), respectively. The resulting solutions were further diluted by mixing one part of 10% Methocel E3 with nine parts of each diluted GRFT sample, yielding a final formulation of 1% Methocel E3 in PBS containing either 15 g/L (high dose) or 7.5 g/L (low dose) GRFT. The final formulations were mixed well, passed through a 0.22 µm filter, aliquoted, and stored at −80 °C.

### 2.6. Propagation and Titration of PRRSV-2 Isolate USA/IN/65239GA/2014 for Pig Study

One PRRSV-2 isolate, USA/IN/65239GA/2014, which was demonstrated to be a virulent strain in a previous study [4], was used as the challenge virus to evaluate the effect of GRFT in pig studies. Propagation and titration of this virus isolate were performed in MARC-145 cells following the previously described procedures [33].

### 2.7. In Vivo Trial

All animal procedures described in this study were reviewed and approved by the Institutional Animal Care and Use Committee (IACUC-22-105, approved on 17 May 2022) and the Institutional Biosafety Committee (IBC-22-059, approved on 17 May 2022) at Iowa State University prior to the initiation of the research.

Fifty-six pigs were weaned at 21 days of age (initial body weight 5.21 ± 0.77 kg; mixed-sex, 1:1) and delivered to the Iowa State University animal facility seven days prior to nasal challenge. Pigs were screened to verify that they were virologically negative for PRRSV, swine influenza A virus, and porcine circovirus 2 and 3 via virus-specific PCRs at the Iowa State University Veterinary Diagnostic Laboratory (ISU VDL). A commercial ELISA kit (IDEXX PRRS X3 Antibody Test, Westbrook, ME, USA) was used to check that all pigs were serologically negative for PRRSV. Each pig was microchipped for monitoring body temperature as described in a previous study [4]. Pigs were blocked by weight and randomly assigned to one of four treatments: non-treated, non-challenged (NT/NC); non-treated, PRRSV-challenged (NT/C); administered the low-dose formulation of GRF and PRRSV-challenged (LDT/C); and administered the high-dose formulation of GRFT and PRRSV-challenged (HDT/C). There were 17 pigs per challenged group (except for the NT/NC with 5 pigs), one group per room, with each room having the same size and condition (Table 1). Pigs were fed a nutritionally adequate swine diet mash and had free access to water. Standard animal enrichments were provided in each room. The room temperature was set in the range of 27–29 °C.

Details of the experimental design and treatments are given in Table 1. From −3 pre-challenge through 10 DPC, Groups 1 and 2 were mock-treated by receiving 1% Methocel E3 in phosphate-buffered saline (PBS) via intranasal spray, while Groups 3 and 4 received the antiviral compound at the corresponding dose via intranasal spray, twice daily. At 0 DPC, Group 1 received virus-negative medium as an unchallenged control, whereas Groups 2 through 4 were challenged with a PRRSV 1-7-4 L1A isolate USA/IN/65239GA/2014 [4] via intranasal (2 mL/nostril) inoculation at a dose of 10^5^ TCID50/pig. All pigs were euthanized and necropsied at 42 DPC.

During the trial, pigs were monitored daily for clinical signs of PRRSV, including lethargy and anorexia. Microchip temperatures were recorded once daily in the morning. Pigs were weighed individually at −7, −3, 0, 10, 14, 21, 28, 35, and 42 DPC (negative values indicate days pre-challenge) to calculate average daily gain (ADG). Blood samples were collected individually from pigs at −7, 0, 2, 4, 7, 10, 14, 21, 28, 35, and 42 DPC and processed to obtain serum. Serum samples were analyzed via a commercial RT-qPCR assay (VetMAX PRRS NA/EU 2.0, Thermo Fisher Scientific, Waltham, MA, USA) at the ISU VDL to determine the PRRSV viremia level.

On 42 DPC of the study (corresponding to 70 days of age), all pigs were euthanized and necropsied for tissue sampling and disease evaluation. The gross lung lesions associated with PRRSV infection were scored by a board-certified pathologist based on the calculated percentages of affected lung lobes as previously described by Halbur PG et al. [34]. Lung tissue samples were fixed in 10% neutral buffered formalin and routinely stained with hematoxylin and eosin. The degree of interstitial pneumonia was evaluated independently by two board-certified veterinary anatomic pathologists based on a scale of 0–4 as previously described [34]. Scores were defined as follows: 0, no microscopic lesion; 1, mild, multifocal interstitial pneumonia; 2, moderate, multifocal interstitial pneumonia; 3, moderate, diffuse interstitial pneumonia; and 4, severe, diffuse interstitial pneumonia.

### 2.8. Statistical Analysis

Data was analyzed by the Kruskal–Wallis test for non-parametric data. Multiple comparisons of interest (treatment vs. controls) were then performed. The false discovery rate (FDR) for multiple comparisons was controlled at 5% with an adjusted significance level set at 5% (*p* < 0.05). All statistical analyses for the in vitro-generated data were conducted in Graph Prism Software (version 10).

Pigs were the experimental unit for the in vivo trial. Data were analyzed by one-way ANOVA, using the Fit Model platform of JMP 14.0. All viral load data were analyzed on a log-transformed scale. Differences between treatment means were determined using Tukey’s test. A *p*-value of <0.05 was considered statistically significant, whereas 0.1 > *p* > 0.05 was considered a statistical tendency.

## 3. Results

### 3.1. GRFT Mitigates PRRSV-Associated Cytopathic Changes in MARC-145 Cells

**Figure 1:**
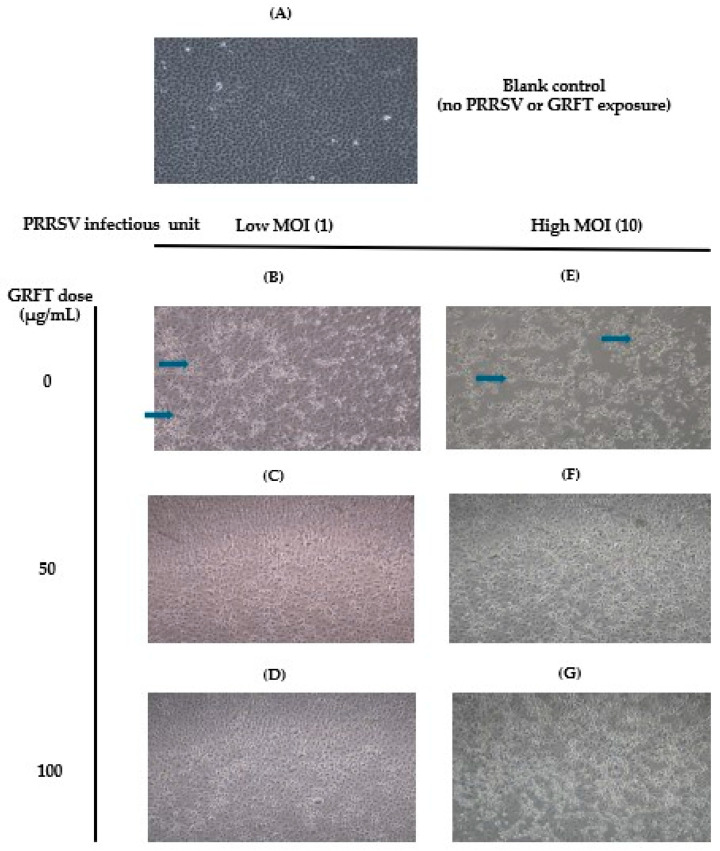
Representative images showing reduced cytopathic effects in GRFT-pre-treated PRRSV-infected MARC-145 cells. (**A**) uninfected, untreated, and monolayers (control treatment); (**B**) cells infected with a low MOI of PRRSV, untreated; (**C**) cells infected with a low MOI of PRRSV, pre-treated with 50 μg/mL GRFT; (**D**) cells infected with a low MOI of PRRSV, pre-treated with 100 μg/mL GRFT; (**E**) cells infected with a high MOI of PRRSV, untreated; (**F**) cells infected with a high MOI of PRRSV, pre-treated with 50 μg/mL GRFT; (**G**) cells infected with a high MOI of PRRSV, pre-treated with 100 μg/mL GRFT. Blue arrows indicate the altered morphology characteristic of infected cells. Representative bright-field images (40×).

Representative bright-field images illustrating PRRSV-associated cytopathic effects (CPE) in MARC-145 cells, with or without GRFT pre-treatment, are shown in Figure 1. Uninfected control cells (Figure 1A) exhibited a uniform, intact monolayer with typical epithelial morphology. In contrast, PRRSV-infected cells without GRFT treatment (Figure 1B,E) displayed marked cytopathic changes, including extensive cell rounding, disruption of the monolayer, and cell detachment (indicated by blue arrows).

Pre-treatment with GRFT visibly mitigated these virus-induced morphological changes. At the lower infection level, GRFT treatment resulted in partial preservation of monolayer integrity (Figure 1C), with reduced cell rounding and detachment compared with untreated, infected controls. The higher GRFT dose provided more pronounced protection, with cell morphology closely resembling that of uninfected controls (Figure 1D). At the higher infection level, similar qualitative effects were observed: untreated, infected cells showed substantial cytopathic damage (Figure 1E), whereas GRFT-treated cells (Figure 1F,G) exhibited reduced CPE and improved monolayer continuity, with greater preservation evident at the higher GRFT dose. These observations are qualitative but were repeatedly observed across different experimental replicates.

### 3.2. GRFT Dose-Dependently Inhibits PRRSV Infection in MARC-145 Cells

**Figure 2:**
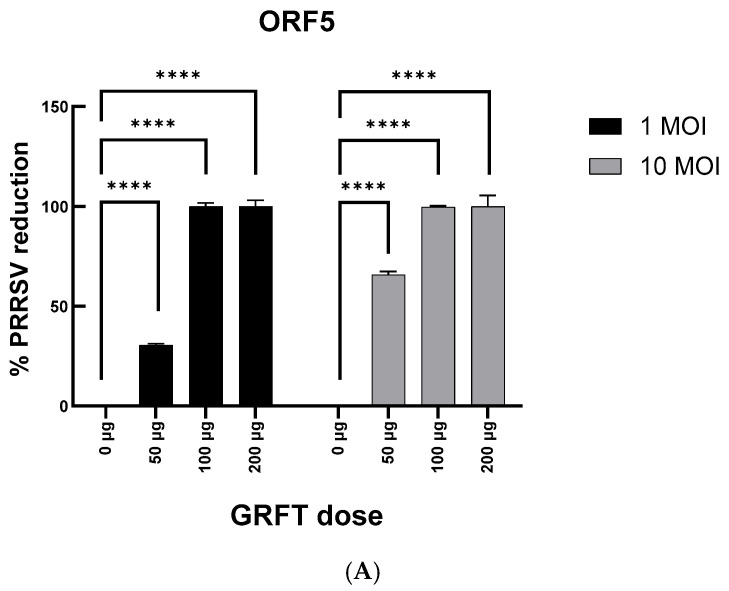

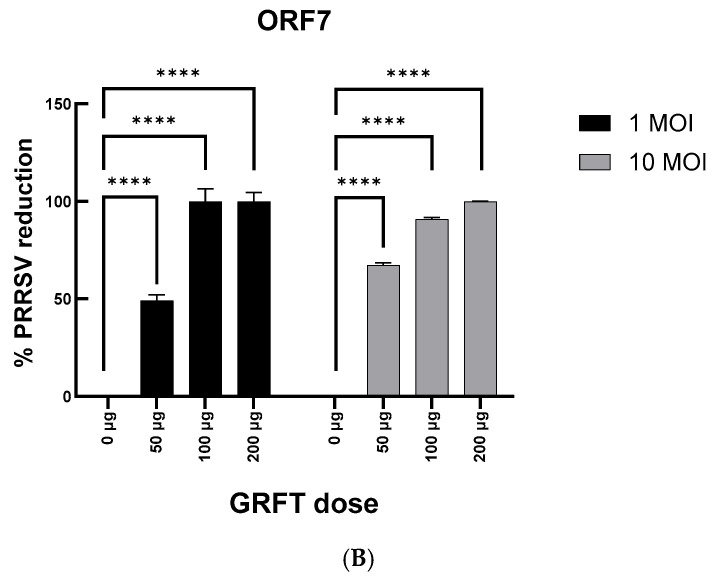
Effect of GRFT pre-treatment of PRRSV-infected MARC-145 cells on the abundance of viral RNA, as quantified via RT-qPCR, using genomic targets (**A**) ORF5 and (**B**) ORF7. Percent PRRSV reduction was calculated relative to untreated controls from ΔCt-normalized expression values. Bars represent mean values with associated SE bars (*n* = 6). **** *p* < 0.0001. MOI, multiplicity of infection.

qRT-PCR analysis of viral RNA collected from PRRSV-infected MARC-145 cells, pre-treated or not with GRFT, revealed robust GRFT dose-dependent reductions in viral RNA abundance at both infection levels (Figure 2). At an infection level of 1 MOI, pre-treatment of cells with 50 µg/mL of GRFT partially inhibited viral replication, resulting in a 30.6 ± 1.4% reduction in PRRSV RNA levels compared with the untreated control (*p* < 0.0001) when ORF5 was used as the RT-qPCR target, and a 49.2 ± 6.8% reduction in PRRSV RNA levels (*p* < 0.0001) when ORF7 was used as the RT-qPCR target, while 100 µg/mL and 200 µg/mL of GRFT achieved almost 100% suppression of viral RNA levels compared with the untreated control (*p* < 0.0001) using either RT-qPCR target. Similar results were obtained at an infection level of 10 MOI. Across both genomic targets, GRFT pre-treatment of cells achieved a consistent inhibitory effect with minimal variability across biological replicates (indicated by the small standard error bars).

### 3.3. Reduced Early Viremia in GRFT-Treated Pigs Following PRRSV Challenge

A rise in the body temperature of PRRSV-challenged pigs was observed during the first five days following challenge (Supplementary Figure S1). This, together with the detection of PRRSV RNA in serum, indicates that the viral challenge was successful. Body temperature returned to normal in treatment HDT/C as early as 14 DPC, compared with 23 DPC in treatment NT/C.

**Figure 3:**
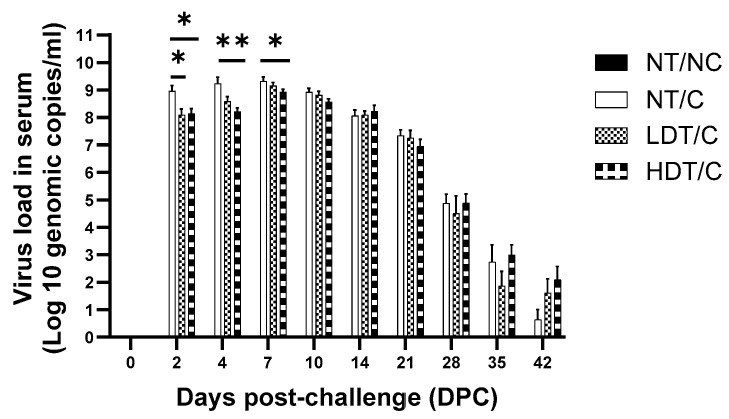
Serum PRRSV viral loads of pigs following PRRSV challenge, with and without GRFT treatment at low or high doses. Values shown are means with associated SE bars (*n* = 5 in NT/NC and *n* = 17 in all other treatments). NT/NC: non-treated, non-challenged; NT/C: non-treated, PRRSV-challenged; LDT/C: treated with the low-dose formulation (7.5 g/L) of GRF and PRRSV-challenged; HDT/C: treated with the high-dose formulation (15 g/L) of GRFT and PRRSV-challenged. * *p* < 0.05, ** *p* < 0.01.

The PRRSV viral loads in the serum of pigs following infection, as determined by RT-qPCR, are presented in Figure 3 by treatment. PRRSV was undetectable in the control treatment NT/NC. The serum viral load of pigs in treatment HDT/C was lower than that of pigs in treatment NT/C on days 2, 4 and 7 post-challenge (day 2: Log_10_ 8.1 genomic copies/mL ± 0.2 vs. 9.0 ± 0.2, *p* < 0.05; day 4: Log_10_ 8.2 ± 0.1 vs. 9.1 ± 0.2, *p* < 0.01; and day 7: Log_10_ 8.9 ± 0.2 vs. 9.3 ± 0.2, *p* < 0.05). These differences correspond to 87.4% reduction in circulating viral RNA on days 2 and 4 and a 60.2% reduction on day 7 relative to NT/C pigs (Supplementary Table S2).

Pigs in the low-dose GRFT treatment group (LDT/C) exhibited a significant reduction in serum viral load compared with NT/C on day 2 post-challenge (log_10_ 8.1 ± 0.2 vs. 9.0 ± 0.2, *p* < 0.05), corresponding to an approximately 87.4% reduction in circulating viral RNA. On day 4 post-challenge, the mean serum viral load in the LDT/C group was log_10_ 8.6 ± 0.2, which was numerically lower than that observed in NT/C pigs (log_10_ 9.1 ± 0.2). By day 7 post-challenge, serum viral loads in LDT/C pigs (log_10_ 9.2 ± 0.1) were comparable to those in the NT/C group (log_10_ 9.3 ± 0.2), indicating that the early reduction in viremia observed with low-dose GRFT treatment was not maintained at later time points.

Notably, the reduction in viremia was observed only during the period of GRFT administration (days 3 to 10 post-challenge), with no sustained reduction detected thereafter. This finding is consistent with a mode of action in which GRFT limits viral entry and early replication rather than eliminating infection. Accordingly, the observed effects represent a transient suppression of early viremia rather than viral clearance.

### 3.4. The Effect of GRFT on Lung Lesions Following PRRSV Challenge

**Figure 4:**
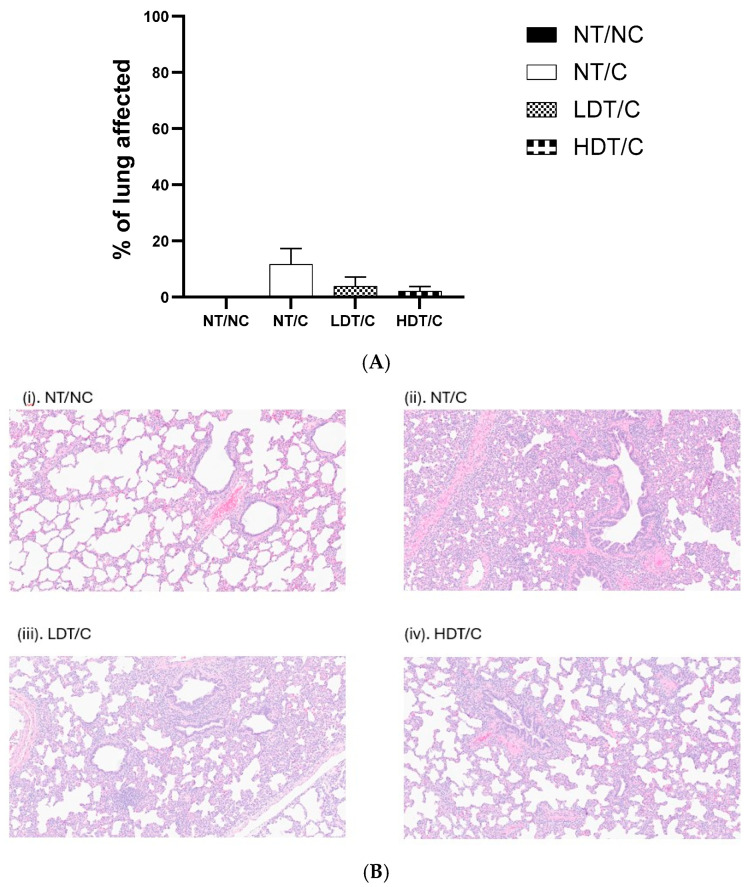

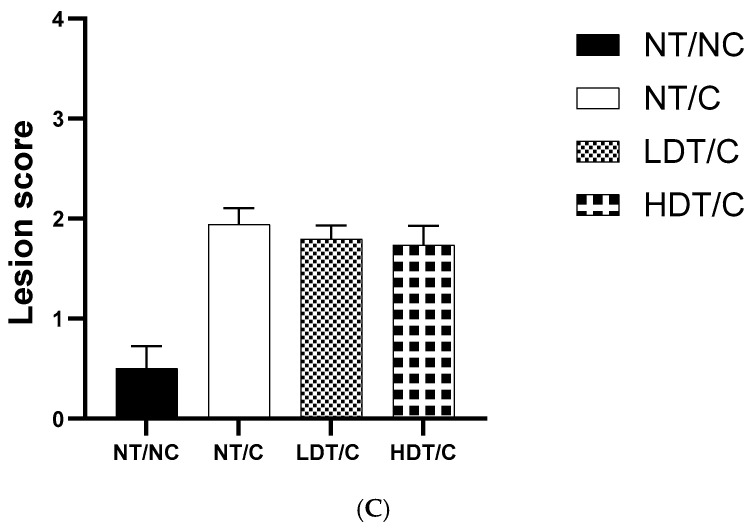
Lung lesion scores in pigs at 42 days following PRRSV challenge, with and without GRFT treatment at low or high doses. (**A**) Percentage of lung gross changes. (**B**) Microscopic images of lungs from different treatment groups taken at 42 DPC (H&E staining). (**i**) Negative control lung with lack of interstitial changes (Score 0). (**ii**) Lungs from a PRRSV-infected pig that were not given treatment. There are multifocal areas of moderate expansion of the alveolar septa and the peribronchiolar interstitium by mononuclear cells (Score 2). (**iii**,**iv**) Lungs from PRRSV-infected pigs given GRFT. There are multifocal areas of mild to moderate expansion of the alveolar septa and peribronchiolar interstitium by mononuclear cells (Scores 1–2). (**C**) Microscopic interstitial lung scores (0–4). Data are means with associated SE bars (*n* = 5 in NT/NC, *n* = 17 in all other treatments). NT/NC: non-treated, non-challenged; NT/C: non-treated, PRRSV-challenged; LDT/C: treated with the low-dose formulation (7.5 g/L) of GRFT and PRRSV-challenged; HDT/C: treated with the high-dose formulation (15 g/L) of GRFT and PRRSV-challenged.

GRFT treatment did not result in statistically significant differences in gross or microscopic lung lesion scores following PRRSV challenge (Figure 4). Lung lesion severity was comparable among PRRSV-challenged groups, and observed numerical differences did not reach statistical significance.

### 3.5. The Effect of GRFT on Mortality Following PRRSV Challenge

All pigs challenged with PRRSV exhibited higher mortality rates than unchallenged pigs (Table 2). Mortality was numerically lower in GRFT-treated groups compared with untreated PRRSV-challenged pigs; however, due to the small group size and low number of events, these differences were not statistically evaluated and should be interpreted descriptively.

### 3.6. The Effect of GRFT on Growth Performance Following PRRSV Challenge

**Figure 5:**
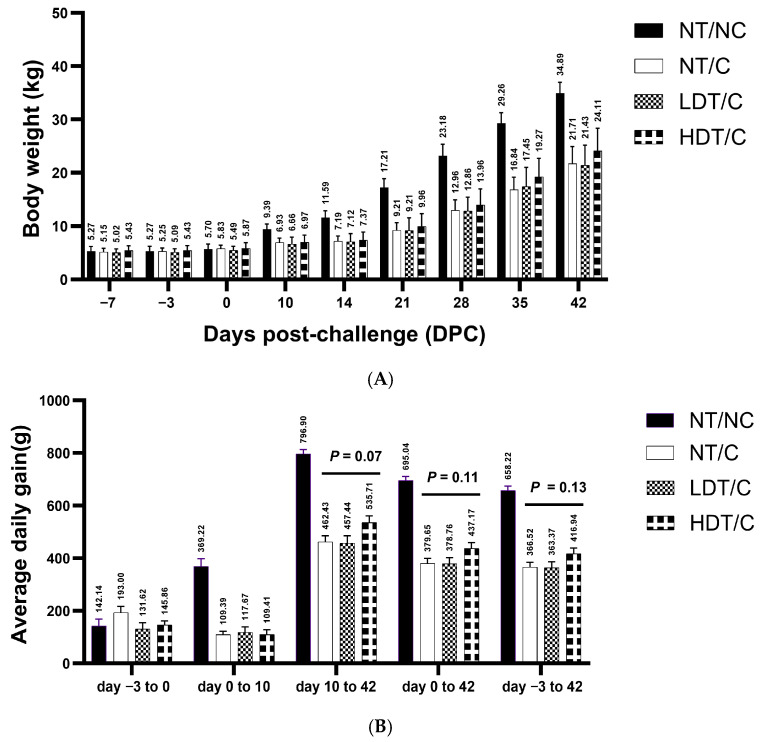
Growth performance over 42 days following PRRSV challenge, with and without GRFT treatment at low or high doses. (**A**) Body weight (BW); (**B**) average daily gain (ADG). Data are means and associated SE error bars (*n* = 5 in NT/NC and *n* = 17 in all other treatments). NT/NC: non-treated, non-challenged; NT/C: non-treated, PRRSV-challenged; LDT/C: treated with the low-dose formulation (7.5 g/L) of GRF and PRRSV-challenged; HDT/C: treated with the high-dose formulation (15 g/L) of GRFT and PRRSV-challenged. *p*-values between pairs of treatments, obtained via one-way ANOVA followed by Tukey’s HSD, are reported where they were near significant.

The effect of treatment on body weight (BW) and ADG is shown in Figure 5. From day 10 post-challenge onwards, all PRRSV-challenged pigs exhibited reduced BW compared with unchallenged controls. GRFT-treated pigs showed numerical but not statistically significant increases in BW and ADG compared with untreated PRRSV-challenged pigs. By the end of the study (day 42 post-challenge), mean BW remained lower in all PRRSV-challenged groups relative to unchallenged controls. Observed differences in BW and ADG among challenged groups did not reach statistical significance and are therefore reported descriptively. Furthermore, confirmation in larger, independently replicated studies would be required to draw firm conclusions.

## 4. Discussion

The challenge of controlling PRRSV infection in swine arises from its genetic diversity, immune evasion capacity that limits vaccine cross-protection, and early attachment/entry events that offer multiple opportunities to establish infection [35,36]. In this context, glycan-binding lectins represent a mechanistically distinct antiviral modality compared with vaccines. Glycan-binding lectins target conserved regions of enveloped viruses—namely surface glycosylation—rather than antigenic epitopes, making them candidates to be exploited therapeutically, as has been discussed in the literature [25,29]. In the present study, we evaluated the efficacy of GRFT using complementary in vitro and in vivo models to determine its potential as an antiviral agent against PRRSV in commercial swine production.

The in vitro studies demonstrated that GRFT pre-treatment substantially reduced PRRSV RNA replication in MARC-145 cells and was associated with reduced viremia after intranasal administration in vivo in pigs. The lung pathological changes and growth performance in GRFT-treated pigs did not differ significantly from controls, with only numerical differences or trends observed. The observed suppression of PRRSV RNA replication in MARC-145 cells following treatment with GRFT is broadly consistent with previous in vitro observations of Li et al. [20]. Li et al. showed that recombinant GRFT exerted saccharide-dependent antiviral activity against PRRSV in MARC-145 cells and mapped the primary effect to the adsorption stage, with no measurable effect on penetration [30]. It was concluded that this was consistent with a mechanism in which lectin binding to virion-associated glycans interfered with early virus–cell interactions [30]. While the present study did not experimentally distinguish viral adsorption from penetration, the pre-exposure design and observed reduction in viral RNA replication at both low (MOI 1) and high (MOI 10) infection doses are compatible with an entry-proximal reduction in infection efficiency, in line with the adsorption-blocking model described by Li et al. [30] and Li et al. [35]. Notably, we observed concordant dose–response patterns from GRFT pre-treatment on viral RNA replication when either of the two commonly used PRRSV genomic regions was targeted in the RT-qPCR (ORF5 and ORF7). This concordant response indicates a strong antiviral signal that was evident across independent detection loci.

Despite the similar overall beneficial effect of GRFT on PRRSV infection in vitro between the present study and in Li et al. [30], the magnitude of the quantitative effects differed and should be interpreted in the context of differences in experimental design and readouts. At an MOI of 10 and a GRFT dose of 4 μg/mL, Li et al. reported a reduction in infected MARC-145 cells of approximately 70%, whereas at the same MOI and a GRFT dose of 100 or 200 μg/mL, we observed almost 100% suppression of viral RNA in cells. Li et al. [30] quantified infection primarily by immunofluorescence of infected cells and by RT-qPCR with different primer targets, whereas our primary endpoint was ΔCt-normalized qRT-PCR of ORF5 and ORF7 expressed as the percentage of viral RNA reduction relative to the control. Infected-cell percentages reflect a discrete cellular outcome, while qRT-PCR integrates both the fraction of infected cells and the average intracellular viral RNA burden, which may accentuate differences when inhibition affects early infection efficiency versus later amplification. In addition, Li et al. [30] included a virion pre-incubation with the GRFT step and tested infection at MOIs in the range of 5–10, whereas the present study focused on testing the effect of MARC-145 cell pre-treatment with GRFT followed by virus infection at MOI levels of 1 and 10. Such design differences can shift the balance between neutralization in solution and interference with attachment at the cell surface. Differences in GRFT preparations and viral strains may also affect the results, and strain-dependent glycosylation patterns on envelope proteins could plausibly influence lectin binding and functional inhibition [25,30]. Notwithstanding this, our findings reinforce that GRFT inhibits PRRSV in vitro while highlighting that potency estimates are context-dependent on assay modality, exposure design, and viral strain.

A principal advance of the present study over the existing knowledge is the observation of a beneficial effect of GRFT in vivo. While the experimental design was small-scale (comprising 5 replications in the control treatment and 17 in the experimental treatments), high-dose (15 mg/pig/day) intranasal GRFT was associated with significantly lower serum viral RNA loads during the early period (first 7 days) post-challenge and earlier normalization of body temperature compared with untreated, challenged controls. These results indicate an early in vivo antiviral effect. An early suppressing effect on viremia is consistent with the broader PRRSV antiviral strategy framework, which identifies viral entry and early replication events as tractable intervention points before systemic amplification begins [23,24]. However, it was notable that later endpoints in our study—including lung lesion scores at necropsy, mortality, and growth performance over the 42 days post-challenge period —showed only numerical differences or trends between treatments. Notably, GRFT administration was limited to the first 10 days post-challenge. As such, any antiviral effect of GRFT would be expected to primarily influence early infection dynamics rather than later disease outcomes measured after treatment cessation.

The limited differences observed in lung lesion scores and growth performance are likely influenced by multiple factors. First, PRRSV-associated lung lesions are known to resolve over time, with pathological changes typically becoming mild by 5–6 weeks post-infection, which is consistent with the findings at 42 days post-challenge in this study. As such, treatment-related differences in lung pathology may have been more evident at earlier stages of infection. In addition, the relatively small group sizes used in this study and the inherent inter-animal variability in PRRSV disease progression may have reduced the statistical power to detect modest differences in downstream pathological and performance outcomes at later time points. Taken together, these considerations suggest that the present in vivo study should be regarded as preliminary and primarily informative for evaluating early infection dynamics, as reflected by the observed reduction in early viremia. Further studies could assess earlier post-challenge time points and examine the impact of dose level and dosing frequency (single versus multiple administration) to better define the conditions under which GRFT may influence both virological and pathological outcomes. Nevertheless, in the tested setting, the early antiviral effects of GRFT did not translate into significant downstream effects. Further larger-scale interventions are therefore needed to confirm whether a long-term suppression of infection is observed and what effect this has on health and growth performance at the whole animal level. It has been acknowledged in the wider PRRSV literature that downstream outcomes can be sensitive to antiviral delivery efficiency, exposure kinetics, and the magnitude of early viral control required to shift later pathology and performance measures, requiring further research [36].

Evidence from non-PRRSV respiratory virus systems provides additional context for intranasal GRFT as a topical antiviral approach. O’Keefe et al. [28] demonstrated that intranasal GRFT conferred an in vivo benefit in a lethal SARS-CoV mouse model and linked its activity to binding of the viral spike glycoprotein and inhibition of entry. This mode of effect demonstrated that mucosal delivery of GRFT can yield measurable antiviral effects for enveloped respiratory viruses [28]. Translationally, Nabeta et al. [31] reported a human Phase 1a/1b clinical trial design for intranasal Q-GRFT emphasizing safety, tolerability, and pharmacokinetics in nasal/nasopharyngeal matrices. While these studies do not address PRRSV directly, they support the mechanistic plausibility of intranasal GRFT delivery and suggest that local exposure and residence time are key variables [28,31]. This may be particularly relevant to explain why early viremia effects in our pig study were significant while later outcomes remained as trends or non-significant.

Our findings are consistent with the adsorption-blocking model described by Li et al. [30], but direct confirmation using our specific GRFT preparation and assay context is needed to strengthen causal inference. Time-of-addition experiments and adsorption/penetration separation assays, combined with glycan competition tests (e.g., with mannose), would determine whether the antiviral activity observed here is similarly saccharide-dependent and adsorption-dominant. Such studies would also clarify whether additional effects observed by Li et al. [30], such as reducing cell-to-cell spread, are also evident using our GRFT preparation and associated test conditions. In vivo, measuring GRFT concentrations and antiviral activity in nasal and/or oral fluids would help link dose to local exposure and guide optimization of dosing frequency, formulation characteristics, and treatment window for PRRSV challenge settings.

## 5. Conclusions

In conclusion, this study has demonstrated that an engineered recombinant GRFT preparation with improved protease stability, produced in an in vitro *Bacillus subtilis* expression system, substantially reduced PRRSV entry in swine lung epithelial (MARC-145) cells in vitro in a dose-dependent manner; GRFT achieved near-complete suppression of ORF5 and ORF7 viral RNA in MARC-145 cells. In addition, intranasal administration of GRFT was associated with a reduction in early PRRSV viremia in young pigs, although this effect was confined to the early phase of infection. Together, these findings support glycan-targeting lectins such as griffithsin as a potential complementary antiviral approach for PRRSV control in swine. Further studies are warranted to elucidate whether the mechanism of effect of this GRFT preparation is saccharide-dependent and adsorption-dominant, and further in vivo studies are needed to guide optimization of dosing frequency and timing.

## Figures and Tables

**Table 1:** Experimental design of the in vivo trial in young pigs.

Treatment No.	Treatment Name ^1^	No. Pigs (*n*)	GRFT Antiviral Treatment (Administered Twice Daily from 3 Days Pre-Challenge to 10 DPC)	PRRSV Challenge
1	NT/NC	5	Intranasal spray of 1% Methocel E3 in PBS (2 mL/nostril)	Intranasal spray of PBS with carrier (2 mL/nostril)
2	NT/C	17	Intranasal spray of 1% Methocel E3 in PBS (2 mL/nostril)	Intranasal spray of PRRSV at a dose of 10^5^ TCID_50_ per pig (delivered as 2 mL/nostril)
3	LDT/C	17	Intranasal spray of low-dose GRFT formulation (2 mL/nostril)	Intranasal spray of PRRSV at a dose of 10^5^ TCID_50_ per pig (delivered as 2 mL/nostril)
4	HDT/C	17	Intranasal spray of high-dose GRFT formulation (2 mL/nostril)	Intranasal spray of PRRSV at a dose of 10^5^ TCID_50_ per pig (delivered as 2 mL/nostril)

^1^ NT/NC: non-treated, non-challenged; NT/C: non-treated, challenged; LDT/C: administered with the low-dose formulation (7.5 g/L) of GRF and PRRSV-challenged; HDT/C: administered with the high-dose formulation (15 g/L) of GRFT and PRRSV-challenged. GRFT, griffithsin; PBS, phosphate-buffered saline; PRRSV, porcine reproductive and respiratory syndrome virus; TCID, tissue culture infectious dose.

**Table 2:** Mortality over 42 days following PRRSV challenge, with and without GRFT treatment at low or high doses.

Treatment	Mortality, *n*/*N* (%)
NT/NC	0/5 (0%)
NT/C	4/17 (23.6%)
LDT/C	2/17 (11.8%)
HDT/C	3/17 (17.6%)

NT/NC: non-treated, non-challenged; NT/C: non-treated, PRRSV-challenged; LDT/C: treated with the low-dose formulation (7.5 g/L) of GRF and PRRSV-challenged; HDT/C: treated with the high-dose formulation (15 g/L) of GRFT and PRRSV-challenged.

## Data Availability

The original contributions presented in this study are included in the article/Supplementary Materials. Further inquiries can be directed to the corresponding authors.

## Supplementary Materials

The following supporting information can be downloaded at: https://www.mdpi.com/article/10.3390/microorganisms14051098/s1, Table S1. Primer sequences and housekeeping genes used in RT-qPCR; Figure S1. Change in body temperature before, during, and post-challenge PRRSV, in pigs pre-treated with or without griffithsin at two different doses; Figure S2. Effect of griffithsin (GRFT) pre-treatment on PRRSV RNA abundance in PRRSV-infected blood-derived macrophages; Table S2. Percent reduction in serum PRRSV viral load relative to NT/C.

## Abbreviations

The following abbreviations are used in this manuscript:

ADG	Average daily gain
BW	Body weight
CPE	Cytopathic effects
CTLs	C-type lectins
DPC	Days post-challenge
FBS	Fetal bovine serum
GAPDH	Glyceraldehyde-3-phosphate dehydrogenase
GRFT	Griffithsin
HDT/C	High-dose GRFT-treated, challenged
LDT/C	Low-dose GRFT-treated, challenged
FDR	False discovery rate
MEM	Minimum essential medium
MOI	Multiplicity of infection
NT/C	Non-treated, challenged
NT/NC	Non-treated, non-challenged
ORF	Open reading frame
PBS	Phosphate-buffered saline
PFU	Plaque-forming unit
PRRSV	Porcine reproductive and respiratory syndrome virus
PS	Penicillin–streptomycin
TCID50	50% tissue culture infectious dose
RT-qPCR	Reverse-transcription quantitative polymerase chain reaction
