# Supplementary material for: Lectin-Based Antiviral Strategies for Porcine Reproductive and Respiratory Syndrome Virus 2 Infection: Griffithsin Suppresses Viral Replication In Vitro and Reduces Early Viremia In Vivo
Source: Microorganisms. 2026 May 12;14(5):1098. doi: 10.3390/microorganisms14051098 (PMC13210354; doi:10.3390/microorganisms14051098)
Supplement: Supplementary file 1 [file microorganisms-14-01098-s001.zip › microorganisms-4241751-supplementary.pdf]

Supplementary Materials

Table S1. Primer sequences and housekeeping genes used in RT-qPCR.

Gene name	Forward primer sequence (5′-3′)	Reverse primer sequence (5′-3′)
Target genes in PRRSV virus		
PRRSV_GP5_ORF5	GGTCGCCCGTCATCA TAGAG	GTTGCCACGGAACC ATCAAG
PRRSV_CP_ORF7	CGGGTACATGACTTT CACGC	CAAACGGCATCTGG AGGTGA
Housekeeping gene		
GADPH	CTGAACGGGAAGCTC ACTGG	GCCTGCTTCACCAC CTTCTT

Figure S1

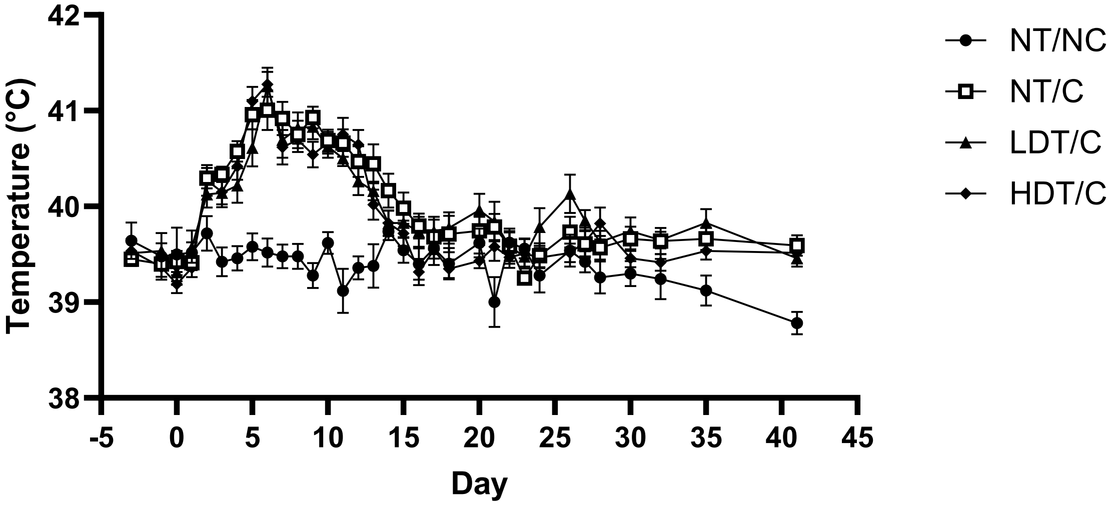

Figure S1. Change in body temperature before, during and post-challenge with PRRSV, in pigs pre-treated with or without GRFT at two different doses. [1]NT/NC: non-treated, non-challenged; NT/C: non-treated, PRRSV-challenged; LDT/C: treated with the low dose formulation (7.5 g/L) of GRFT and PRRSV-challenged; HDT/C: treated with the high dose formulation (15 g/L) of GRFT and PRRSV-challenged.

Material and Methods

Isolation and differentiation of peripheral blood-derived macrophages

Peripheral blood mononuclear cells (PBMCs) were isolated from porcine blood by density gradient centrifugation using Ficoll (SOP137). All washes were performed with wash buffer (DPBS + 2% FBS). PBMCs were counted and resuspended at 5×10^7 cells/ml in wash buffer. Cells were incubated for 20 min at room temperature with a cocktail of primary antibodies (1:100 each): mouse anti-pig CD3, mouse anti-human CD79a, and mouse anti-pig granulocytes. After washing, cells were resuspended in MACS buffer (DPBS + 0.5% BSA + 2 mM EDTA) and incubated with anti-mouse IgG microbeads on ice for 15 min. Magnetic separation was performed using MACS columns according to the manufacturer's instructions. The unlabeled (negative) cell fraction was collected, centrifuged, and resuspended in TC medium (RPMI 1640 + 10% FBS + 1% penicillin-streptomycin).

Cells were seeded at 1×10^6 cells/well in 6-well plates or 3×10^5 cells/well in 24-well plates in TC medium supplemented with 50 ng/ml M-CSF. One well was lysed immediately after seeding as Day 0 control. Cells were cultured at 37°C, 5% CO_2 for 4 days. Macrophage differentiation was verified by morphological assessment and by measuring the expression of macrophage marker genes CD163 and SIGLEC1 by qPCR. On Day 4, cells were activated by replacing the medium with fresh TC medium containing 10 ng/ml LPS and 100 ng/ml IFN-γ for 24 h. On day 5, cells were infected with GRFT + virus or virus alone for 1 hour at 37 °C. After the 1-hour incubation, the virus was removed, and the cells were further incubated for 24 hours at 37 °C. Following incubation, the cells were washed and lysed with ice-cold RA1 lysis buffer. The lysates were stored at -80 °C until RNA extraction and qPCR analysis.

Griffithsin (GRFT) Dose-Dependently Inhibits PRRSV Infection in PBMCs-Derived Macrophages

RT-qPCR analysis of viral RNA from PRRSV-infected blood-derived macrophages pre-treated with GRFT revealed a dose-dependent inhibitory effect on viral replication (Figure S2). At an infection level of 1 MOI, pre-treatment with 10 μg/mL GRFT resulted in modest and variable reductions in PRRSV RNA abundance, with a 29.9 ± 9.0% reduction when ORF5 was used as the RT-qPCR target and a 26.3 ± 11.8% reduction when ORF7 was used. In contrast, increasing the GRFT concentration to 50 μg/mL led to near-complete suppression of viral RNA levels, resulting in reductions of 98.9 ± 0.1% (ORF5) and 98.9 ± 0.1% (ORF7). Treatment with 100 μg/mL or 500 μg/mL GRFT further achieved essentially complete inhibition of PRRSV replication, with viral RNA reduced by more than 99% across both genomic targets.

A similar inhibitory trend was observed at an infection level of 10 MOI. While 10 μg/mL GRFT produced limited and variable inhibition (18.2 ± 19.7% for ORF5 and 20.8 ± 14.2% for ORF7), higher GRFT concentrations resulted in pronounced antiviral effects. Specifically, 50 μg/mL GRFT reduced PRRSV RNA levels by 62.6 ± 3.5% (ORF5) and 62.4 ± 5.1% (ORF7), whereas 100 μg/mL and 500 μg/mL GRFT achieved robust suppression of viral RNA abundance, exceeding 93% reduction across both targets. Overall, GRFT pre-treatment consistently inhibited PRRSV replication in PBMCs-derived macrophages in a dose-dependent and target-independent manner, with minimal variability observed at higher GRFT concentrations.

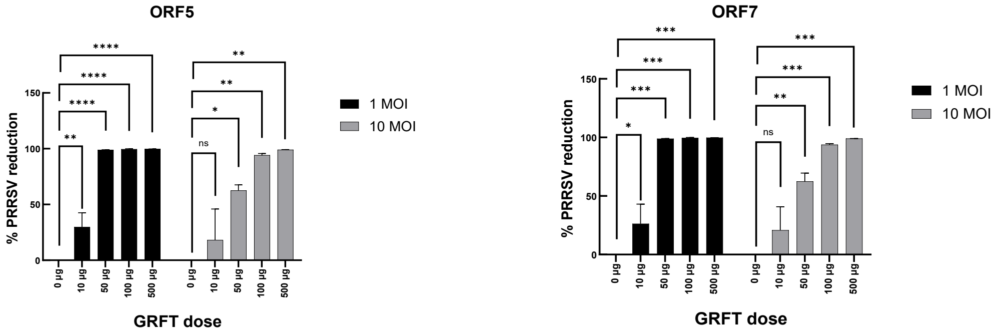

Figure S2. Effect of griffithsin (GRFT) pre-treatment on PRRSV RNA abundance in PRRSV-infected blood-derived macrophages. Viral RNA levels were quantified by RT-qPCR using the genomic targets ORF5 (left panel) and ORF7 (right panel) following infection at 1 MOI or 10 MOI. Percent PRRSV reduction was calculated relative to untreated controls from ΔCt-normalized expression values. GRFT pre-treatment resulted in a clear dose-dependent reduction in viral RNA levels, with near-complete suppression observed at higher GRFT concentrations for both genomic targets. Bars represent mean values with associated standard error (SE) bars from two biological replicates. Statistical significance is indicated as *$P < 0.05$, **$P < 0.01$, ***$P < 0.001$, ****$P < 0.0001$; ns, not significant. MOI, multiplicity of infection.

Table S2. Percent reduction in serum PRRSV viral load relative to NT/C

Day post-challenge	NT/C (log_{10} copies/mL)	HDT/C (log_{10} copies/mL)	HDT/C % reduction vs NT/C	LDT/C (log_{10} copies/mL)	LDT/C % reduction vs NT/C
Day 2	9.0	8.1	87.4%	8.1	87.4%
Day 4	9.1	8.2	87.4%	8.6	69.7%
Day 7	9.3	8.9	60.2%	9.2	28.3%

Percent reduction was calculated by converting log_{10} viral loads to absolute values and expressing the reduction relative to the NT/C group. LDT/C values for days 4 and 7 represent the mean of 17 individual measurements.
